# Development and psychometric evaluation of waste separation beliefs and behaviors scale among female students of medical sciences university based on the extended parallel process model

**DOI:** 10.1186/s12199-020-00849-6

**Published:** 2020-04-16

**Authors:** Aazam Abbasi, Marzieh Araban, Zahra Heidari, Masoumeh Alidosti, Fereshteh Zamani-Alavijeh

**Affiliations:** 1 Student Research Committee, School of Health, Isfahan University of Medical Sciences, Isfahan, Iran; 2 Department of Health Education and Promotion, Public Health School, Ahvaz Jundishapur University of Medical Sciences, Ahvaz, Iran; 3 Department of Biostatistics and Epidemiology, School of Health, Isfahan University of Medical Sciences, Isfahan, Iran; 4 Department of Health Education and Promotion, School of Health, Isfahan University of Medical Sciences, Hezar Jarib, Avenue, Isfahan, 81676-36954 Iran

**Keywords:** Psychometrics, Validity, Reliability, Waste Separation, Student

## Abstract

**Background:**

The increasing production of un-recycled waste is a great threat to public health. Therefore, assessment and measurement of people’s beliefs and perceptions with regard to these threats can contribute to the development of suitable educational messages promoting waste separation behaviors. This study aimed to carry out the scale development and psychometric evaluation of behaviors and beliefs associated with waste separation among female students.

**Method:**

This methodological research was performed in 2019. The primary questionnaire was developed based on the assessment of waste separation beliefs and behaviors based on the extended parallel process model. Afterwards, to confirm the content and face validity of the research tool, the opinions of 14 faculty members and certain students were asked for, respectively. In order to assess the construct validity of the questionnaire, exploratory factor analysis was performed based on the data collected from 386 female students in Isfahan University of Medical Sciences, Iran. The internal and external reliability of the tool was determined through estimating Cronbach’s alpha and test-retest based on intraclass correlation (ICC) index, respectively.

**Results:**

The mean age and academic semester of the students were 22 ± 1.9 years and 5.58 ± 2.6, respectively. The primary version of the questionnaire was designed with 65 items; one item was omitted during the content validity process. Construct validity with factor analysis technique yielded nine dimensions including 64 items with a factor loading above 0.3. The overall reliability of the research tool was confirmed at Cronbach’s alpha of 0.87. Furthermore, the ICC of the entire questionnaire was 0.89.

**Conclusion:**

According to the results of the study, the final 64-item questionnaire could be used by various researchers to assess waste separation beliefs and behaviors considering suitable psychometric features.

## Introduction

During the recent decades, the growing production of solid wastes has led to serious environmental problems threatening the public health [1]. Waste production has risen tenfold in the past century [2]. Currently, billion metric tons of municipal solid waste (MSW) are annually produced. This value is expected to be doubled in the next decade even without population growth [3, 4]. In most developing countries, the increasing production of urban solid wastes has led to a phenomenon known as “waste siege” [5]. In Iran, there are concerns regarding the risk of diseases caused by the environmental ramifications associated with unhealthy dumping of solid wastes in open spaces around cities [6]. Poor waste management causes environmental degradation, soil and water contamination, different types of infectious or chronic diseases, and reduced quality of life [7, 8].

Widespread public participation in adopting waste separation at source is among the most important strategies in waste management and a key to success in this area [9, 10]. As such, the determinants of which must be identified [3]. For instance, previous studies have introduced age, gender, social norms, economic incentives, access to facilities, attitude, social support, self-efficacy, and perceived effectiveness as factors related to adopting waste separation behaviors [10–13]. It has further been reported that informing the public and different types of visual messages affect the separation intention [14] and promotion of waste separation behavior, respectively [15].

Health education theories and models such as health belief model (HBM) and theory of planned behavior (TPB) have been applied in some studies to specify the factors affecting waste separation behaviors [3, 7, 8, 16, 17]. According to HBM, understanding people’s perceptions regarding the dangers of non-segregated waste is critical for studying its associated behavior. In a study based on HBM, perceptions such as perceived severity, self-efficacy, perceived benefits, perceived susceptibility, perceived barriers, and knowledge affected a sanitary disposal of waste [17]. Other factors might further be involved in adopting this behavior. A frequent criticism of the TPB is related to its reliance on rational reasoning for the determinants of behavior while overlooking other such factors as subconscious effects and emotions. In general, waste separation-related behaviors may not ensue from rational deliberation [3].

**Fig. 1:**
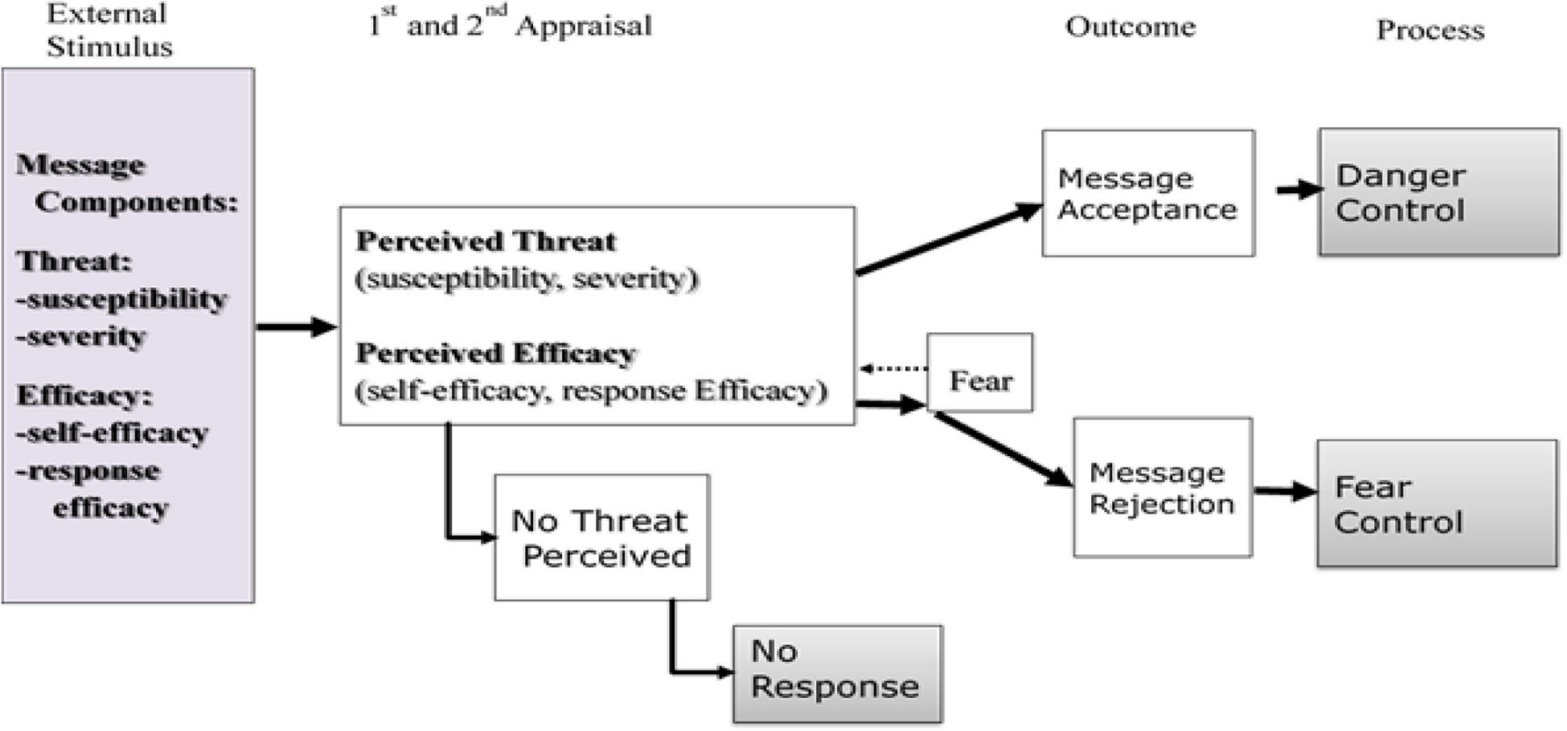
The extended parallel process model (EPPM)

Different factors are possibly involved in the formation of effective waste separation behavior. Therefore, a more comprehensive framework to fathom these behaviors is highly required [3]. In this regard, the extended parallel process model (EPPM) can be a more applicable and suitable framework since it not only considers the role of perception raised in HBM, but also deals with the role of attitude and intention raised in TPB in the formation of behavior. This method further takes into account the role of emotions and the response to these emotions (Fig. 1) [18].

This model is also able to measure the impact of received messages and people’s reactions in this regard. According to EPPM, recognizing the endangerment to health by a serious threat following educational messages, being ensured of the effectiveness of solutions in dealing with this threat, and the ability to employ preventive behaviors and ensure their positive impacts would more possibly alter the attitude, intention, and health beliefs of people with regard to danger control. On the other hand, high perception of threat and low self-efficacy create the undesirable feeling of fear, where the main response is to deal with the fear and not the danger. In this case, danger-related messages are either avoided or deemed worthless [18, 19]. Therefore, it is necessary to provide appropriate tools for measuring each of these factors or evaluating the outcomes of educational messages in different groups of society.

Despite all the good and remarkable features of waste separation and recycling, this behavior has not been fully institutionalized in people’s lives [20]. As the most important strategy for separating recyclables, public participation can in fact guarantee the successful implementation of recycling [7]. Some groups are more influential among people, even serving as a reference for others. Young people, especially students, are more likely to accept new behaviors. Other groups in society become more influenced by the behaviors of this group [9, 21]. Therefore, by recognizing the factors leading to behavior and conducting educational interventions to promote health behaviors in youth, particularly students, one can promote health-related behaviors in the community.

Moreover, some experts hold that women are more concerned about environment than men, and in Iranian societies specifically, they have a stronger connection with environment [22]. Moreover, women in the family might be willing to perform waste management in the home and kitchen [23, 24]. According to the above descriptions, in the future, female students probably play a greater role in promoting waste segregation principles in the family.

All in all, students in general and female students in particular are an influential group in the society; however, the assessment of factors affecting waste separation using a valid and reliable measure in students has not been well fathomed [25, 26]. Therefore, the present study was carried out for the development and psychometric evaluation of waste separation beliefs and behaviors in female students in Isfahan University of Medical Sciences, Iran, using EPPM.

## Methods

This methodological research was performed on 386 female students residing in the dormitories of Isfahan University of Medical Sciences, Iran, during June to August 2019.

### Scale development process

The primary version of the questionnaire was developed in a preliminary study in EPPM framework (Fig. 1), based on the review of scientific texts [13, 18, 19, 27–30] obtained by extended search in Google Scholar, Cochrane Library, PsychInfo, Scopus, PubMed, Magiran, SID, and Science Direct.

In this regard, the authors prepared item pool to measure the EPPM perceptual and behavioral variables and perceived barriers. For this purpose, the authors asked for oral opinions of six experts in the fields of health education (*N* = 4), environmental health (*N* = 1), and biostatistics (*N* = 1). The process culminated in designing a scale containing 65 items in 12 constructs.

The first five dimensions of the questionnaire for assessing perceptions included “perceived sensitivity” (eight items), “perceived severity” (ten items), “response efficiency” (four items), “self-efficacy” (seven items), and “perceived barriers” (five items). These items were scored with alternatives of “strongly agree,” “agree,” “undecided,” “disagree,” and “strongly disagree.”

The sixth dimension of the questionnaire, “fear” induced by the news related to waste risks, was assessed based on mood adjectives, including frightened, tense, nervous, anxious, uncomfortable, and nauseous with six items, using alternatives of “extremely,” “very,” “moderately,” “not at all,” and “never.”

The seventh to ninth dimensions were used to assess three responses of individuals in terms of fear control processes. The measurement items of these responses were prepared in three sections, namely “message minimization” (three items) and “feeling manipulated” (four items), with alternatives of “extremely,” “very,” “moderately,” “not at all,” and “never,” and “defensive avoidance” (three items), with alternatives of “always,” “very often,” “sometimes,” “rarely,” and “never.”

The 10th–12th dimensions assessed the three responses of individuals regarding risk control, meaning adaptive responses [18]. The dimensions included “attitude” (four items), with alternatives of “extremely,” “very,” “moderately,” “rarely,” and “never”; “intention” (four items), with alternatives of “since now,” “over the next 2 weeks,” “over the next month,” “over the next 6 months,” and “over the next year”; and “behavior” (six items), with alternatives of “always,” “very often,” “sometimes,” “rarely,” and “never.”

The selected alternatives were scored from 1 to 5, with higher scores indicative of positive beliefs and behaviors in line with waste separation.

### Validity

Content, face, and construct validity methods were used to determine the validity of the questionnaire.

#### Content validity assessment

For quantitative assessment of content validity, 14 experts in the fields of health education (*N* = 9), environmental health (*N* = 4), and statistics (*N* = 1) were asked to select one of the alternatives of “essential,” “useful but not essential,” and “not necessary” for each item of the questionnaire and estimate the content validity ratio (CVR) based on their responses. Afterwards, items receiving a CVR above 0.51 based on Lawshe’s table were maintained (*P* < 0.06) [31]. In the next stage, the opinions of the same experts were asked for regarding the questionnaire items in a 4-point Likert scale in terms of (1) relevance, (2) simplicity, and (3) clarity. Subsequently, content validity index (CVI) was measured for each item [32]. In this respect, items that received a CVI above 0.79 were considered acceptable [33].

#### Face validity assessment

Face validity was quantitatively determined by asking for opinions of 20 students regarding the importance level of each item and its alternatives in a 5-point Likert scale from “not important at all” (score = 1) to “very important” (score = 5). Afterwards, the impact score of each item was specified by estimating the result of multiplying the importance coefficient by relative frequency; items scored ≥ 1.5 were kept in the questionnaire.

#### Construct validity assessment

The factor structure of the questionnaire was explored using the exploratory factor analysis (orthogonal varimax rotation procedure) among 386 students. Factors were remained for further analysis based on their interpretation and eigenvalues on the scree plot. In this study, we retained factors with eigenvalues > 1.5 as cutoff and factor-item loading values higher than 0.3, which could result in more interpretable factors and explain sufficient amounts of overall variation. The data suitability for factor analysis was guided through the Kaiser-Meyer-Olkin (KMO) measure of sample adequacy (values > 0.7) and Bartlett’s test of sphericity (*P* < 0.05). The final extracted factors were labeled based on the loaded items in each factor. The factor score for each subscale (factors) was computed through summing up the items multiplied by related loading and assigned to each participant [34].

### Reliability assessment

To assess the reliability, a test-retest was carried out on a group of 30 students. The questionnaires were completed by students over two stages with a 1-week interval; ICC was estimated to evaluate external reliability. Cronbach’s alpha was estimated in order to determine the internal consistency and reliability of the questionnaire [35].

To evaluate test-retest reliability, we computed the intraclass correlation (ICC) coefficient using two-way mixed model along with 95% confidence interval. A coefficient of more than 0.70 was considered as excellent stability [36]. Internal consistency was assessed using Cronbach’s α coefficient. The values between 0.70 and 0.95 were conventionally considered as satisfactory internal consistency [36].

### Ethical considerations

The research was approved by the Ethics Committee of Isfahan University of Medical Sciences with the code of IR.MUI.RESEARCH.REC.1398.165. Notably, the subjects were ensured of the confidentiality terms regarding their personal information, and a written informed consent was obtained prior to the research.

## Results

The mean age of the students was 22.01 (1.945) years, and the mean academic semester was 5.58 (2.600).

The CVI and CVR were above 0.79 and 0.51, respectively, confirming the content validity of the questionnaire. However, item 24 was eliminated from the “Perceived barriers” section although there was no need for modifying the item to remain in the questionnaire due to the presence of a similar item (Additional file 1).

### Construct validity

Construct validity was assessed through the use of exploratory factor analysis (EFA) with varimax rotation extracting nine factors from the scale, namely “perceived threat,” “efficiency,” “fear control: reacting against the communicator or message,” “behavior,” “intention,” “attitude,” “fear,” “fear control: defensive avoidance,” and “perceived” accounting for 16.72%, 9.04%, 7.70%, 6.02%, 5.95%, 4.91%, 4.65%, 3.84%, and 3.58% of total variance, respectively. A KMO value of 0.878 and *P* < 0.05 for Bartlett’s test confirmed the data viability for factorability. Table 1 provides the factor loadings of the nine factors extracted from EFA on the 64 items of the measure.

**Table 1:** Factor analysis and reliability results for discovery of main factors of questionnaire for evaluation of waste segregation beliefs and behaviors

Abbreviation	Rotated component matrix									
	Item	Component								
		Perceived threat	Efficacy	Reacting against the communicator or message	Behavior	Intention	Attitude	Fear	Defensive avoidance	Perceived barriers
Seve25	1	.810								
Seve26	2	.802								
Seve21	3	.771								
Susc18	4	.764								
Susc16	5	.762								
Seve24	6	.760								
Seve28	7	.743								
Susc15	8	.742								
Susc20	9	.741								
Susc19	10	.731								
Seve23	11	.726								
Susc17	12	.706								
Seve29	13	.699								
Seve22	14	.687								
Susc14	15	.672								
Seve27	16	.639								
Susc13	17	.619								
Seve30	18	.388								
Eff43	19		.839							
Eff44	20		.813							
Eff41	21		.797							
Eff42	22		.786							
Eff45	23		.659							
Eff46	24		.601							
ResEff39	25		.555							
ResEff40	26		.532							
ResEff37	27		.472							
Eff47	28		.459							
ResEff38	29		.360							
FCR56	30			.797						
FCR55	31			.778						
FCR54	32			.766						
FCD57	33			.763						
FCD58	34			.750						
FCD59	35			.727						
FCD60	36			.644						
PRA69	37				.792					
PRA70	38				.761					
PRA73	39				.742					
PRA71	40				.716					
PRA68	41				.638					
PRA72	42				.464					
INT77	43					.877				
INT74	44					.841				
INT76	45					.829				
INT75	46					.823				
ATT61	47						.690			
ATT63	48						.634			
ATT64	49						.623			
ATT62	50						.617			
fear51	51							.767		
fear52	52							.746		
fear50	53							.660		
fear53	54							.651		
fear49	55							.511		
fear48	56							.469		
FED66	57								.835	
FED67	58								.790	
FED65	59								.685	
barr32	60									.647
barr31	61									.539
barr34	62									.467
barr35	63									.436
barr33	64									.370
	Eigenvalue	14.434	6.960	5.493	3.706	2.216	2.012	1.858	1.693	1.570
	Variance %	22.553	10.875	8.582	5.790	3.463	3.144	2.903	2.645	2.453
	Variance cumulative %	16.722	25.760	33.462	39.484	45.431	50.337	54.985	58.827	62.408
		Extraction method: principal component analysis Rotation method: varimax with Kaiser normalization								
		a. Rotation converged in 9 iterations								

### Internal and external reliability assessment results

The reliability was confirmed for the whole questionnaire and each factor after performing factor analysis through Cronbach’s alpha. In this regard, Cronbach’s alphas of the first, second, third, fourth, fifth, sixth, seventh, eighth, and ninth factors were estimated at 0.942, 0.791, 0.897, 0.709, 0.896, 0.733, 0.853, 0.850, and 0.879, respectively. Cronbach’s alpha of the entire tool was further calculated at 0.876, thereby confirming the internal reliability of the tool. On the other hand, the ICC was estimated at 0.675, 0.816, 0.975, 0.608, 0.721, 0.736, 0.566, 0.858, 0.753, and 0.889 for the first, second, third, fourth, fifth, sixth, seventh, eighth, and ninth factors, and the entire tool, respectively. This confirmed the external reliability of the tool (Table 2). Table 2 also shows the distributions and the main results based on the instrument among participants.

**Table 2:** The distributions and the main results based on the instrument among participants

	The number of items	Possible range	Mean score (SD)	Cronbach’s α	ICC (95% CI)	95% confidence interval	
						Lower bound	Upper bound
Perceived threat	18	18–90	79.00 (9.30)	0.942	0.675	0.421	0.831
Efficacy	11	11–55	47.00 (5.92)	0.791	0.816	0.648	0.908
Reacting against the communicator or message	7	7–35	14.00 (6.02)	0.897	0.975	0.949	0.988
Behavior	6	6–30	18.00 (5.63)	0.709	0.608	0.323	0.792
Intention	4	4–20	12.00 (6.24)	0.896	0.721	0.492	0.857
Attitude	4	4–20	19.00 (2.77)	0.733	0.736	0.515	0.865
Fear	6	6–30	18.00 (5.15)	0.853	0.566	0.264	0.767
Defensive avoidance	3	3–15	12.00 (2.59)	0.850	0.858	0.724	0.930
Perceived barriers	5	5–25	17.00 (3.31)	0.879	0.753	0.543	0.874

## Discussion

The first step in designing an intervention to change any behavior is to prepare the proper tools for assessing the behavior and former perception level of individuals [18, 37]. The primary goal of this research was the development and psychometric evaluation of tools used to evaluate the perception towards the risk of waste accumulation in environmental and public health. Another objective of the novel tool was to assess the feeling and reaction of individuals when exposed to educational messages and their manner of adopting waste separation behaviors. Therefore, we performed our study in EPPM framework. A list of 65 relevant items was prepared following the targeted evaluation of valid texts, related articles, and questionnaires. Estimation of CVR and CVI showed that 64 items were approved. Only one item acquired an insufficient level of necessity (barr36) by the panel of experts.

Exploratory factor analysis was carried out in order to assess the construct validity. To be more conservative and not delete an item by merely asking the expert panel, all 65 items were entered at this stage. Factor analysis was run with a number of different factors (between 8 and 13 factors), and the adequacy of each was further evaluated. Finally, a 9-factor model was considered as the final model through deleting the item number barr36 due to the low factor loading (0.186). As such, this model was identified as the most interpretable model with 64 items and 9 factors.

### Perceived threat

The first exploratory factor was “perceived threat,” which included 18 items of perceived sensitivity and perceived severity associated with waste risks. It is often difficult to separate the items in measuring the perceived sensitivity and perceived severity. The integration of these two constructs has been formerly reported [38]. According to previous studies and based on EPPM, people have to perceive the severe risk of accumulation and lack of waste separation [39]. Measuring the perception of risk severity and level of vulnerability can help anticipate and justify preventive behaviors [37]. In addition, the assessment of information and risk associated with environmental behaviors has been proposed as an environmental health literacy construct [40]. As such, appropriate tools must exist to measure this variable in several studies and assess preventive behaviors and risk control, including waste separation [41, 42].

### Perceived efficacy

The second extracted factor was “efficiency” with 11 items. This factor was obtained by integrating the items of two dimensions in the primary questionnaire, including the efficiency of waste separation behavior in reducing hazards and self-efficacy, which is understanding one’s own ability to separate all wastes. In addition to assessing the perceived threat in the EPPM framework, it is crucial to estimate efficiency [43] in order to determine its role in predicting such healthy behaviors as waste separation. The next step after perceiving threat is to improve perceived self-efficacy, which is effective in preventive behaviors [43, 44]. A study showed that individuals performed waste separation behavior if they believed that separating waste increased the recycling of valuable products, protected recyclable materials against pollution, and increased their reuse [45]. According to EPPM, it seems that the change in attitude, intention, and behaviors such as waste separation is higher in terms of perceiving the related threat and positively assessing the efficacy of recommended behaviors. In this regard, it is extremely crucial to have standard tools to confirm or reject such type of hypothesis [43, 46].

### Fear

A construct discovered in this study was “fear,” encompassing six items. Fear of negative consequences motivates people to change unwanted behavior patterns. Behaviors can be successfully changed by use of different techniques of fear-based messages [46]. In a study, some families pointed out the significant benefits obtained from recycling metal objects; however, they did not separate plastic bottles due to the fear of negative consequences associated with their reuse [41]. According to Witte, ineffective fear can prevent behavior change and the promotion of healthy behaviors. Therefore, evaluation of fear and its determinants based on EPPM can contribute to the promotion of effective behaviors [18, 37, 47].

### Reacting against the communicator or message

The fourth extracted construct was “reacting against the communicator or message” because this 17-item part of the questionnaire assessed the reactions of students to waste warning messages. According to Witte, individuals make such reactions to control fear [18]. This part of the questionnaire was obtained from integrating the items related to measuring two reactions proposed by Witte, including “message minimization” and “feeling manipulated,” which showed a type of maladaptive reaction to risk messages or the communicator. To control their fear, individuals tend to make the risk messages seem worthless and exaggerated [19, 37, 48]. Cismaru asserted that people react against warning messages when they perceive a high level of threat but believe in their lack of ability to reduce the associated risks [47]. On the other hand, if there is a high perception of threat and low perception of efficacy, they only engage in fear control instead of adopting the correct behavior and reducing risk. Therefore, there must be a balance between threat and efficacy in order to promote behaviors such as waste separation. In this respect, designing tools and measuring such variables can guide efficacy enhancing interventions.

### Defensive avoidance

Another construct was “defensive avoidance” which, similar to the fourth factor, is a maladaptive reaction of people to control fear, not danger. However, this construct is different in that people who perceive fear but believe in their inefficient behaviors avoid warning messages in any way possible [19, 37, 48]. In the present research, the developed tools assessed the reaction of students to avoiding the reception of messages associated with the risk of wastes and their separation. As previously reported, people often react in a way to reduce their fear (avoiding reading, listening, or talking about the subject) as a consequence of risk messages. According to EPPM, via suitable tools, we can more focus on the prevention of unfavorable impacts of messages on the audience and engaging them in fear control process and lack of behavioral changes [47].

### Attitude

The sixth extracted factor was “attitude” comprising four items. To design a successful program for recycling, we must improve the attitude of people towards environmental issues to promote proper behaviors in this respect [28]. Fabian pointed out that a positive attitude is one of the strongest constructs that more efficiently explains the intention to conduct recycling behaviors [49]. This scholar further posited that the attitude of students towards proper waste recycling behavior was improved by education, which can affect the behavior of families with regard to intergenerational influences [50]. The first step in designing educational programs on waste recycling is to consider the public attitude [41, 51]. The adaptive threat control reactions of individuals can be assessed through the use of suitable tools in order to measure their attitude, intention, and behavior [18, 43].

### Intention

In the present tool, one of the extracted constructs was “intention,” comprised of four items. Evaluation of intention can show the success or failure of using EPPM [18, 19, 37, 43, 48] and indicate the threat control reaction in individuals. Moreover, it can properly anticipate the actual behaviors of people [52, 53]. Instead of measuring intention, Saphores et al. evaluated and compared the variable of willingness to recycle electronic waste and reported its association with behavior [54]. On the other hand, Wang et al. measured intention and reported its correlation with waste recycling behavior [16]. Another research showed the necessity of identifying the attitude and intention to conduct tourism promotion behaviors in the environment [55]. In a research, Echegaray and Hansstein observed that recognition and perception of complications associated with electronic waste on environment play a significant role in intention [49]. Nonetheless, the direct association between perceiving threat and intention of conducting a behavior has been formerly hypothesized [19]. Accordingly, it seems crucial to design a proper tool for measuring intention and its determinants and related behaviors.

### Behavior

The eighth six-item construct extracted in the tool was “behavior.” According to Witte in EPPM, risk control-related behaviors indicate one’s adaptive responses [18]. Therefore, waste separation behavior in the present research was an adaptive response to controlling the threat of actual risks caused by lack of management. In other words, the key objective is to specify whether the threat and efficiency components in educational messages have been effective or not. According to this model, educational intervention improves the behaviors of people if risk-decreasing methods and behaviors are emphasized in educational messages as far as fear is concerned [56]. The pro-environmental behavior (PEB) has been deployed as a solution to promoting eco-friendly model through measuring, promoting, and teaching waste separation behaviors [57]. A correct waste separation behavior in youth can be created through increasing their perception and concerns regarding waste topic and simple and practical solutions [24]. However, valid tools are required to assess such behaviors.

### Perceived barriers

The ninth construct was “perceived barriers” with five items. This construct was added to the default model following the suggestion of the team of authors and approval of the expert panel. This construct measures barriers to waste separation from the perspective of students. Given the time-consuming nature of the recycling process, this behavior is less employed by manufacturers due to barriers such as weak infrastructures and compulsory rules for regulating long-term programs [49]. According to Echegaray and Hansstein, perceived barriers could prevent behaviors as an independent variable despite the increase in perceived threats and efficiency in a study [49]. Other preventive factors affecting environment-related behaviors include lack of sufficient space in different places [41] and paucity of suitable conditions to recycle paper [52]. In a study, 47.8% of the subjects were eager to conduct waste separation behaviors if such barriers were removed and separate containers were available for waste recycling [41]. Therefore, it is recommended that this item be measured in addition to the model’s construct when using EPPM.

### Research limitations

- A major drawback of the present study was the psychometric evaluation of this questionnaire which was conducted only on a group of young individuals. Other age groups (adults and children) should be considered in future studies. Moreover, it is suggested that confirmatory factor analysis be carried out for more validity.
- Not distributing the questionnaire among male students and community residents was also a limitation of the current study.
- Not to do confirmatory factor analysis is a limitation that should be assessed in future results.
- A randomized controlled trial is further recommended to assess the responsiveness to the change in the questionnaire.
- According to the EPPM, the external stimuli, message processing, and outcomes are determinants of a given behavior or a recommended response [18]. As such, the questionnaire is not applicable for measuring the external stimuli. However, it can assess the following: (1) message processing (appraisals), which includes “perceived threat,” “perceived efficacy,” and “fear”; (2) outcomes that are (i) “danger control” that could be investigated by 3 parts of the current questionnaire consisting of “attitudes,” “intention,” and “behaviors” and (ii) “fear control” that are assessable by those items relating to “defensive avoidance” and “reacting against the communicator or message.”

## Conclusion

According to the results of the present study, various researchers can employ the final questionnaire with 64 items and nine factors to identify waste separation behaviors and beliefs and evaluate the impact of educational intervention owing to its suitable psychometric properties.

## Supplementary information

**Additional file 1.** Results of quantitative content and face validity of the questionnaire item.

## Data Availability

All data will be available without restriction upon reasonable request.

## Abbreviations

EPPM: Extended parallel process model
WHO: World Health Organization
