# Supplementary material for: Development and psychometric evaluation of waste separation beliefs and behaviors scale among female students of medical sciences university based on the extended parallel process model
Source: Environ Health Prev Med. 2020 Apr 16;25:12. doi: 10.1186/s12199-020-00849-6 (PMC7164206; doi:10.1186/s12199-020-00849-6)
Supplement: Supplementary file 1 — Additional file 1. Results of quantitative content and face validity of the questionnaire item. [file 12199_2020_849_MOESM1_ESM.doc]

***Additional file 1. Results of quantitative content and face validity of the questionnaire items***

| **CVR** | **CVI** | **Cronbach's Alpha if Item Deleted** | **Std. Deviation** | **Mean** | **Item** | **Row** | **Factor** |
| --- | --- | --- | --- | --- | --- | --- | --- |
| 1.00 | .93 | .873 | .890 | 4.37 | I will be exposed to environmental diseases if there is no waste separation. | 1 | **Perceived sensitivity** |
| 0.71 | .96 | .873 | .563 | 4.60 | Environmental destruction endangers my health. | 2 |
| 0.71 | .93 | .875 | .572 | 4.50 | Animal disease and death caused by lack of waste recycling will endanger my health. | 3 |
| 1.00 | 1 | .872 | .814 | 4.40 | The next generation is at environmental risks caused by lack of waste separation. | 4 |
| 0.86 | 1 | .872 | .621 | 4.60 | The nature around us is at risk due to the lack of waste recycling. | 5 |
| 1.00 | .95 | .873 | .626 | 4.57 | Water and soil contamination caused by lack of waste separation endangers my health. | 6 |
| 0.86 | .86 | .872 | .626 | 4.57 | The high level of waste mismanagement shows the threat to the health of my family members and me. | 7 |
| 1.00 | .86 | .873 | .571 | 4.47 | Accumulation of waste endangers my life. | 8 |
| 1.00 | .95 | .874 | .563 | 4.40 | Diseases caused by lack of waste separation might be deadly. | 9 | **Perceived severity** |
| 0.71 | .86 | .870 | .855 | 4.40 | Diseases caused by feeding on waste-fed products might be deadly. | 10 |
| 0.57 | .93 | .872 | .675 | 4.60 | Long-term waste decomposition (up to hundreds of years) in nature will destroy the future generations. | 11 |
| 1.00 | .95 | .873 | .626 | 4.43 | Waste-contaminated water might be deadly to me and my family members. | 12 |
| 0.86 | .95 | .870 | .711 | 4.33 | Plastic waste accumulation will endanger my life and my children’s life | 13 |
| 0.86 | .98 | .873 | .571 | 4.53 | Diseases caused by waste-contaminated soil will have irreversible consequences for me and my children. | 14 |
| 0.86 | .98 | .874 | .758 | 4.33 | Cancer caused by lack of waste decomposition in nature will have dangerous consequences for me and others. | 15 |
| 1.00 | .98 | .872 | .630 | 4.50 | Animal disease and death caused by feeding on waste will have consequences for me. | 16 |
| 1.00 | .98 | .871 | .837 | 4.30 | Animal disease and death caused by feeding on waste will have consequences for my children. | 17 |
| 0.86 | .88 | .873 | .740 | 4.27 | High costs caused by source loss because of the lack of waste separation will lead to severe poverty in the community. | 18 |
| 1.00 | 1 | .876 | .860 | 3.87 | Sometimes, I forget to recycle waste. | 19 | **Perceived**  **barriers** |
| 0.86 | 1 | .881 | 1.125 | 3.90 | I have insufficient time for proper waste segregation. | 20 |
| 1.00 | 1 | .879 | 1.006 | 3.43 | My roommates do not cooperate in terms of proper waste separation. | 21 |
| 0.86 | .90 | .883 | 1.009 | 2.50 | I do not know how to properly recycle waste. | 22 |
| 0.86 | 1 | .872 | .907 | 4.07 | I am not in the mood to recycle. | 23 |
| 1.00 | .90 | .871 | .568 | 4.43 | Proper waste separation effectively decreases environmental pollution around me. | 25 | **Response Efficacy** |
| 0.71 | .93 | .873 | .675 | 4.40 | Waste separation increases my sense of content. | 26 |
| 0.86 | .95 | .873 | .571 | 4.53 | Proper waste separation is in favor of my country’s economy and booming of related industries. | 27 |
| 0.86 | .93 | .872 | .571 | 4.47 | One of the advantages of proper waste separation is a cleaner environment for the next generation. | 28 |
| 0.86 | .98 | .872 | .571 | 4.47 | I can separate metal cans from other waste. | 29 | **Self-efficacy** |
| 0.86 | .98 | .874 | .450 | 4.73 | I can separate paper from other waste. | 30 |
| 0.86 | .98 | .873 | .556 | 4.37 | I can separate glass objects from other waste. | 31 |
| 0.86 | .93 | .870 | .679 | 4.57 | I can separate plastic objects from other waste. | 32 |
| 0.86 | .95 | .871 | .571 | 4.47 | I can separate bread pieces from other waste. | 33 |
| 1.00 | 1 | .874 | .621 | 4.40 | I can persuade my friends and families to recycle. | 34 |
| 0.86 | .83 | .871 | 1.129 | 4.03 | I can provide facilities for waste separation. | 35 |
| 0.71 | .93 | .876 | .999 | 2.03 | Messages related to the impact of waste on environmental pollution frighten me. | 36 | **Fear** |
| 0.57 | .95 | .880 | .915 | 1.70 | I become tense when I see the pictures of animals died because of waste accumulation. | 37 |
| 0.71 | .81 | .877 | 1.017 | 2.00 | Messages indicating the destruction of natural sources caused by lack of waste separation make me nervous. | 38 |
| 0.86 | .95 | .880 | 1.258 | 2.07 | Messages related to waste segregation make me anxious. | 39 |
| 0.86 | .95 | .879 | 1.155 | 2.10 | Messages related to waste separation make me uncomfortable. | 40 |
| 0.43 | .90 | .875 | 1.516 | 3.33 | Messages related to waste segregation make me nauseous. | 41 |
| 0.86 | .86 | .872 | 1.322 | 3.10 | I believe that messages related to waste separation are exaggerated. | 42 | **Fear Control: Decrease message value** |
| 1.00 | .93 | .868 | 1.317 | 3.30 | I believe that messages related to waste separation are overblown. | 43 |
| 1.00 | .93 | .874 | 1.363 | 2.93 | I believe that messages related to waste separation are overstated. | 44 |
| 0.86 | .93 | .873 | 1.337 | 2.73 | I believe that messages related to waste separation are misleading. | 45 | **Fear Control: feeling Distorting and manipulating the message** |
| 0.57 | .81 | .872 | 1.112 | 2.73 | I believe that messages related to waste separation are distorted. | 46 |
| 0.86 | .79 | .874 | 1.192 | 2.60 | I believe that designers of messages related to waste separation try to pressure me in a certain way. | 47 |
| 0.71 | .81 | .871 | 1.542 | 3.03 | I believe that messages related to waste separation try to manipulate me. | 48 |
| 1.00 | 1 | .876 | .774 | 4.77 | Is waste separation beneficial for me? | 49 | **Attitude** |
| 0.86 | 1 | .874 | .484 | 4.80 | Is waste separation important to me? | 50 |
| 0.86 | .98 | .876 | .535 | 4.70 | Is waste separation pleasurable for me? | 51 |
| 0.86 | 1 | .876 | .583 | 4.73 | Is waste separation favorable to me? | 52 |
| 0.71 | .86 | .871 | 1.432 | 3.47 | I immediately block the passage of a message received on waste separation. | 53 | **Fear Control:**  **Avoiding Defenses** |
| 0.86 | .93 | .871 | 1.224 | 3.87 | I have no willingness to talk about waste-related risks. | 54 |
| 0.86 | .93 | .872 | 1.276 | 3.60 | I avoid seeing waste-related images (movies, posters, and photos). | 55 |
| 1.00 | 1 | .879 | 1.112 | 3.27 | Do you currently separate metal cans from other waste? | 56 | **Behavior** |
| 1.00 | 1 | .875 | 1.159 | 4.03 | Do you currently separate paper from other waste? | 57 |
| 1.00 | 1 | .876 | 1.165 | 3.57 | Do you currently separate glass objects from other waste? | 58 |
| 1.00 | 1 | .875 | 1.073 | 3.57 | Do you currently separate plastic objects from other waste? | 59 |
| 1.00 | 1 | .875 | 1.129 | 3.97 | Do you currently separate bread pieces from waste? | 60 |
| 1.00 | 1 | .879 | 1.042 | 3.53 | Do you currently motivate your friends and families to recycle? | 61 |
| 0.86 | .93 | .873 | 1.042 | 4.13 | I intend to plan for daily waste separation. | 62 | **intention** |
| 1.00 | .93 | .873 | .997 | 4.20 | I intend to recycle waste throughout the day. | 63 |
| 0.71 | .90 | .876 | 1.269 | 3.90 | I intend to recycle all waste. | 64 |
| 1.00 | .93 | .872 | .913 | 4.17 | I intend to prepare all necessary facilities for daily waste separation. | 65 |
